# Supportive supervision for volunteers to deliver reproductive health education: a cluster randomized trial

**DOI:** 10.1186/s12978-016-0244-7

**Published:** 2016-10-03

**Authors:** Debra Singh, Joel Negin, Christopher Garimoi Orach, Robert Cumming

**Affiliations:** 1 Kimanya-Ngeyo Foundation for Science and Education, PO Box 1600, Jinja, Uganda; 2 University of Sydney, School of Public Health, Sydney, Australia; 3 Makerere College of Health Sciences, School of Public Health, Kampala, Uganda

**Keywords:** Community Health Workers, Community Health Volunteers, Supportive supervision, Accompaniment, Maternal and Newborn Health, CHWs, CHVs, Supportive supervision, Empowerment, Pregnancy, Neonatal health

## Abstract

**Background:**

Community Health Volunteers (CHVs) can be effective in improving pregnancy and newborn outcomes through community education. Inadequate supervision of CHVs, whether due to poor planning, irregular visits, or ineffective supervisory methods, is, however, recognized as a weakness in many programs. There has been little research on best practice supervisory or accompaniment models.

**Methods:**

From March 2014 to February 2015 a proof of concept study was conducted to compare training alone versus training and supportive supervision by paid CHWs (*n* = 4) on the effectiveness of CHVs (*n* = 82) to deliver education about pregnancy, newborn care, family planning and hygiene. The pair-matched cluster randomized trial was conducted in eight villages (four intervention and four control) in Budondo sub-county in Jinja, Uganda.

**Results:**

Increases in desired behaviors were seen in both the intervention and control arms over the study period. Both arms showed high retention rates of CHVs (95 %). At 1 year follow-up there was a significantly higher prevalence of installed and functioning tippy taps for hand washing (*p* < 0.002) in the intervention villages (47 %) than control villages (35 %). All outcome and process measures related to home-visits to homes with pregnant women and newborn babies favored the intervention villages. The CHVs in both groups implemented what they learnt and were role models in the community.

**Conclusions:**

A team of CHVs and CHWs can facilitate families accessing reproductive health care by addressing cultural norms and scientific misconceptions. Having a team of 2 CHWs to 40 CHVs enables close to community access to information, conversation and services. Supportive supervision involves creating a non-threatening, empowering environment in which both the CHV and the supervising CHW learn together and overcome obstacles that might otherwise demotivate the CHV. While the results seem promising for added value with supportive supervision for CHVs undertaking reproductive health activities, further research on a larger scale will be needed to substantiate the effect.

## Plain English Summary

To address shortages in the health-care workforce some low- and middle-income countries have drawn on Community Health Volunteers (CHVs) to bridge gaps by offering preventative reproductive health care. Limited by relatively short training they volunteer 5–10 h per week and receive little or no remuneration and poor supervision. In recent years, to make CHV efforts more effective, some countries have chosen to train younger people with higher basic education for 6 months to 2 years as Community Health Workers (CHWs) as full-time members of the health system. Having a system of supportive supervision while desirable has often been of low priority and too costly for most programs. To further increase their reach to address social determinants of reproductive health problems and engage the community, this study is looking at whether one of the roles of CHWs could be to train and supportively supervise CHVs. The study compared the work of ~10 community and self-selected CHVs in four villages who were trained alone versus the same number who were trained and supportively supervised to make home visits to pregnant women and newborn babies by four CHWs over a 1-year period.

### Conclusion

After a year 95 % of all CHVs were retained, with increased numbers of home-visits in all eight villages. Those CHVs that were accompanied significantly increased community participation as seen by the increase in the number of community built hand-washing devices. The CHVs also acted as role models in their community by implementing the strategies they were teaching about.

## Background

Experience has shown that well-trained and supervised part-time Community Health Volunteers (CHVs) and full-time Community Health Workers (CHWs), when supported by a functioning healthcare system, can be effective at improving maternal health, and, even in the context of a weak primary health care system, can improve child and neonatal health outcomes [1].

Making a distinction between these two cadres – CHVs and CHWs – has been helpful for planning purposes as demands on CHWs are becoming greater [2]. The current trend in low and middle- income countries is to attract young people with at least 9 or 10 years of basic education as CHWs [3–5]. These individuals then receive professional training of 6 months to 2 years and are fully integrated into the health system, at times with a career path [3–5]. While CHWs can deliver well-circumscribed clinical and preventative services, including family planning, antenatal care, immunization and integrated Community Case Management (iCCM), close to the community, their time is limited [1, 5, 6]. A role still exists, therefore, for older, respected members of the community, who can extend the reach of the CHWs and engage the community in transformative processes to address the social and cultural determinants of health [1, 6]. These CHVs for the most part, have received less formal and informal health-related education, and because of a number of commitments, are only able to volunteer up to 5 to 10 h per week. The Ethiopian Health Extension Worker (HEW) program utilizes both CHWs and CHVs [2, 7–9]. The two full-time HEWs (or CHWs) serving a population of 5000, train and supervise model families during home visits and group sessions, over a 96-h period as part of their routine work which includes a number of clinical duties [2, 7–9]. Some of those trained become volunteers that engage other community members in community-based change to improve health outcomes [2, 3, 9].

While supervision has been acknowledged [1, 10–14] as important in improving CHV performance, much of the evidence is anecdotal [15]. Challenges for supervision fall into three main categories: low priority, lack of finances [10, 12] and poor quality due to lack of training or guidance of those supervising [5, 13]. As a result, it is not uncommon for CHVs to be trained and then receive only clinic-based, not field-based, supervision [16–18]. Poor roads, lack of fuel and inadequate or costly transport arrangements are frequently stated barriers [10, 12, 16] with motivation of supervisors being distorted when per diems are offered [17, 18]. The World Health Organization describes how “traditionally, many countries have used an authoritarian, inspection or control approach to supervision. This approach is based on the thinking that health workers are unmotivated and need strong outside control to perform correctly.” [19] However, this assumption may be faulty, with a number of studies showing that, despite a lack of remuneration, many CHVs are motivated by knowledge acquisition, community recognition and respect [20–24].

Culturally, supervision may be considered intimidating and disempowering and as such, could be detrimental [12, 13]. Defining the purpose of the supervision and providing appropriate training for those supervising becomes critical in the design and planning of CHV programs.

The Innovations at Scale for Community Access and Lasting Effects (inSCALE) project examined various supervisory options [14] and suggested that the quality of the supervision is more important than the frequency. In a recent review of 22 studies, Rowe and colleagues conclude that few CHV supervisory models have been rigorously tested but there is some indication that supportive approaches, community monitoring [25, 26], and quality assurance and problem solving [13, 14, 27, 28] may be most effective. Involving community and religious leaders and peer support may also positively impact the acceptability and motivation of CHVs [14, 25, 28].

The most supportive approaches are respectful and non-authoritarian [12, 14, 15]. Some programs have found that experienced or senior CHVs or full-time CHWs can be effective supervisors [7, 8, 29–32] and that, with time, intensive initial supervision can be replaced by peer support and less frequent visits [32]. As more full-time CHWs are being trained and integrated into health systems they may be the logical supervisors of CHVs.

In Uganda, Village Health Teams (VHTs) – groups of CHVs – receive an initial training and then intermittent refreshers according to Ugandan Ministry of Health (MoH), District Health or NGO priorities. Situational analyses conducted in 2009 and 2015 found that quarterly centralized meetings were most likely to constitute supervision and that no records of field visits could be identified [17, 18]. There were 22 possible supervisory individuals or bodies, without specific training on when, what and how to supervise VHTs, with programs involving treatment and distribution of commodities more likely to be supervised than those conducting health promotion [18]_._

To help address the gap in understanding of supervisory models, this paper presents the findings of the primary outcomes of a small-scale pair-matched cluster randomized trial (CRT) comparing monthly training of CHVs by CHWs alone, to monthly training plus supportive supervision while on home visits. Specifically the aim of the present study was to use the rigour of a CRT in the evaluation of different approaches to supervision to determine if supportive supervision would improve retention rates, numbers of home visits related to pregnant women and newborn babies, and improve specific outcomes related to hygiene.

## Methods

### Setting

The proof of concept study took place in eight villages in the sub-county of Budondo in Jinja district, East Uganda. The largest government health facility, where the majority of women deliver their babies, is 20 km from the nearest hospital in Jinja town that can offer care, such as blood, oxygen and caesarean sections in obstetric emergencies. Located close to the River Nile, Budondo has a predominantly rural based population, who are mostly subsistence farmers, homemakers, shopkeepers and fishermen. Recent trends in growth of sugarcane as a cash crop has created issues with food insecurity at household level. The main ethnic group is Basoga.

The cluster unit was the village. The researcher resident in Uganda selected the eight study villages, from two parishes. An attempt was made to find pairs of villages according to the distances to main roads and health facilities and the number of households in each arm of the study, as well as if they had received NGO support. All of the villages in the study had previous experience with the Village Health Team (VHT) program, which trained and deployed VHTs, the equivalent of a CHV. The VHT coordinator informed the research team that most of the VHTs/CHVs, while identifying as a VHT member, were inactive from 2010, and therefore at the time of the study. While focusing mostly on preventative health, VHTs/CHVs in Uganda have been trained in a variety of roles depending on the needs of the MoH. It was noted that home visiting was done only in the context of drug or mosquito net distribution. Community members would, at times, visit the VHT/CHV at their home for advice or treatment. Interventions by other organizations were also taken into account, with villages with high levels of input excluded to prevent contamination. There was a buffer zone of at least one village between villages included in the study. Meetings with community leaders were conducted in all villages to explain the study and there were no refusals to join.

### Ethics approval participants and design

The pair-matched cluster-randomized study was undertaken between March 2014 and February 2015. A modified WHO 1988 EPI sampling methodology was used to conduct baseline household surveys in March 2014, with participants being mothers of children under 5 years of age. The CHV coordinator and existing or previous CHVs who knew the village well moved with the research group. A random household was chosen in the centre of the village and a pen was spun to identify the initial direction taken and the sampling took place with every fifth household. If the household did not have a child under the age of five then the household to the right was visited until a child was identified. A total of 216 baseline household surveys conducted. Information on household hygiene and location of delivery were collected, as well as data related to newborn care, breastfeeding, infant feeding, weaning, immunization and family planning practices. Questions about the number of recommended antenatal visits; timing of initial breastfeeding; timing of first immunization; length of exclusive breastfeeding and introduction of solids were included in the post-intervention (*n* = 201) questionnaire.

The names of the paired villages were then sent to the researchers at the University of Sydney who randomly allocated one village in each pair to the intervention group and one to the control (see Table 1). Randomization took place at the end of March 2014, following the baseline household surveys. A follow-up household survey was conducted in February 2015.

**Table 1:** Details of matched pairs of villages in Budondo

Pair	Village	Village type	No. homes	Km HC IV^a^	Other^b^
1	Kyoma East	Control	275	5 km	No
	Kagera-Kidiope	Intervention	205	5 km	No
2	Nakanyoni	Control	383	4 km	1.5 km HCII
	Bwase	Intervention	241	4 km	1 km HCII
3	Kizinga	Control	327	4 km	Private Clinic
	Bususwa	Intervention	237	4 km	1 km HCII
4	Bufula A	Control	300	4.5 km	0.5 km HCII
	Nawangoma	Intervention	191	5 km	2 km HC III

^a^Health Centre II, III, IV - hierarchy of health centres in Uganda

^b^Other Health Centre

The District health management team was informed and provided advice from the beginning of the study. Community leaders, who divided each village into four zones, convened meetings and representatives from each zone attended the meetings. Two or three of the 10 people in each village who became CHVs resided in each zone. Five of the CHVs in each village were selected during the community meeting according to criteria provided (over 18, respected, literate, resident and willing to volunteer) and five CHVs volunteered because of their interest in the study. This latter group will be referred to as self-selected. Self-selected CHVs were included to see how their retention rates, demographic and other characteristics differed from community-selected CHVs.

A total of 82 CHVs were recruited for this study. Demographic information was collected from the CHVs for the purpose of comparing CHVs from intervention and control groups. Seven of the eight study villages had community leaders amongst the CHVs for a total of 26 community leaders: 13 in each arm of the study.

A 2-day initial training was offered to all the CHVs in the study in a centralized location. Ongoing monthly training was offered to the groups of eight to twelve CHVs in their own village from a full-time CHW for two to three hours per month over a 10-month period on topics related to the role of the CHV in providing education through home visits and stimulating community action as a means to improve maternal and newborn health outcomes. Specific topics included village mapping, the importance of attending four antenatal visits, encouraging birth at a health facility, danger signs in pregnancy, birth preparation, early breastfeeding and immunization, newborn care including cord care and kangaroo care for small babies, family planning, use of ORS in diarrhea, hand-washing and how to build tippy taps. Hygiene was considered important to improve newborn outcomes. The educational content was aligned to the MoH priorities. The package was piloted during May to November 2013. Lack of trust of public health interventions was identified during focus groups and additional training materials related to trust building were included to overcome misconceptions in the community about public health interventions. The CHVs received 5000 Ugandan shillings (US$2) per month to cover their transport costs.

The CHVs were asked to volunteer 5 to 10 h per week with a male and female CHV working in pairs so as to have access to the women and men in the family. The CHVs carried MoH flip charts on relevant issues and videos about breastfeeding and danger signs in newborns shown on computers or pico-projectors, which were shown to families in their homes and in antenatal clinics. While the men predominantly worked on environmental health and hygiene, they also engaged men on issues related to antenatal care, birth preparation and danger signs in pregnant women and newborn babies.

Four full-time CHWs were involved in the project. They were paid the equivalent of US$80 per month. One had been a CHV coordinator for 18 years, one was a school-teacher with HIV counseling background and two had no health background. Weekly training for CHWs was provided by the researcher in Uganda and visiting doctors about the topic CHWs would train and supervise the CHVs in during the following month. CHWs reflected together after every visit to the villages and more formally during the weekly CHW training. They also had access to health professionals, the internet and books to answer specific questions that arose.

The supervisory model therefore had the following characteristics: there were four full-time and paid CHWs receiving weekly training who were responsible for training all 82 CHVs. In the early learning phase, all four of the CHWs went together to all eight villages to conduct the training. Within 6 months, their capacity was built and their confidence grew, they divided into a male and female CHW team who trained the groups of four villages with approximately eight to twelve CHVs in their village monthly.

The same CHWs also supervised the 43 CHVs in the intervention villages. Emphasis was placed on developing the relationship between supervising CHWs and CHVs. A gender appropriate CHW did home visits with 4–5 CHVs from two zones at a time, on the topic that had been covered in the previous training. The supervising CHW would model initial introductions and conversations with a family and then allow the CHVs to practice. He or she would also help the CHVs answer challenging questions that arose in the initial months of the intervention. The visit was referred to as accompaniment rather than supervision, during which the CHVs and CHWs learnt together with reflection at the end of the visit to discuss successes and challenges. On comparison with the usual practice of CHV training and supervision in Uganda, the control group received high quality monthly training in their own villages rather than intermittent training and centralized quarterly meetings if supervised.

The following outcomes were expected from the intervention: (a) that supportive supervision would result in higher retention rates amongst the CHVs in the intervention compared to the control group; (b) that CHVs in the intervention villages would make more home visits to all homes in their zones and more home visits to pregnant women and newborn babies; and (c) that homes in the intervention villages would have higher utilization of methods to improve hygiene, including tippy taps, dish-racks, water purification and Oral Rehydration solution (ORS) for diarrhea.

### Statistical analysis

With 80 % power, and a design effect of 2.0, a total of 200 households (25 from each of the eight study villages) – 100 households in the four intervention villages and 100 in the four control villages – was enough to detect a 30 % difference at follow-up in the use of tippy taps and/or household dish-racks, use of purified water, women knowing a CHV and having been visited by them, visits to homes with pregnant women (2 visits) and newborn babies (3 visits in the first week) and use of ORS with diarrhoea. A p value of 0.05 was considered statistically significant.

We calculated means and proportions of the baseline characteristics to identify any major differences between the households and the CHVs in the control and intervention villages. We used an intention to treat approach, with unmatched two sample t-tests, to compare process and outcome variables between the intervention and control arms [33]. This method, proposed by Diehr, takes into account the fact that randomisation was at the village level (cluster), not at the level of individual study subjects.

The researcher in Uganda reviewed data for accuracy and completeness and the research team returned the following day to collect any missing data from the participants. Data were double entered and 10 % of entries re-checked.

## Results

The villages included in the study had populations between 1000 and 2000 people, with differences between pair-matched villages of up 500 people. Villages had similar socio-economic characteristics, access to education, water supply and health facilities (see Table 1). All villages were 4–5 km from the Health Centre IV–the largest government health facility in the sub-county-with three of the four pairs of villages having access to a smaller health facility.

Table 2 provides demographic data from the pre- and post- household surveys. Study participants differed in some respects compared to data from the Demographic Household Survey (DHS) of 2011 for the whole Jinja district. Despite more of the women having attended some years of primary school compared to the DHS, there were fewer that were able to read a sentence. The villages also had a higher Muslim population (approximately 36 %) compared to the percentage in Jinja district (13 %) (see Table 2).

**Table 2:** Characteristics of study subjects in intervention and control villages and Uganda Demographic and Health Survey

	Control		Intervention		DHS 2011
	*N* = 113		*N* = 104		Jinja District
	No.	%	No.	%	%
Mean age	27.4		28.1		
School					
Primary	62	54.9 %	51	45.1 %	50 %
Secondary	33	29.2 %	33	32.4 %	25.1 %
Higher education	2	1.8 %	2	1.9 %	0.9 %
None	16	14.2 %	21	20 %	17.6 %
Literacy					
Can’t read	35	31 %	48	40.9 %	16 %
Partially read	30	26.5 %	18	17.3 %	11.8 %
Can read	47	41.6 %	36	34.6 %	41.3 %
Wrong language	1	1 %	2	1.9 %	1 %
Missing			1	1 %	
Religion					
Muslim	42	37.2 %	37	35.6 %	13 %
Christian	71	62.8 %	67	64.4 %	87 %
Tribe					
Musoga	80	70.8 %	74	71.1 %	
Other	33	29.2 %	30	28.9 %	
Mean no. in house	5.8		5.4		5.1

There were no important differences in the mean age, education level, literacy rates household sizes, wealth indicators or hygiene factors between participants in the control and intervention villages (see Table 2).

### CHV demographic information and retention

The demographic information of the CHVs is provided in Tables 3, 4 and 5. There was no difference in retention rates of CHVs in the control or intervention villages, which was 95 % after 1 year in both groups. The four CHVs that dropped out were self-selected, two each from the control and intervention group, three within the first 2 months of the study. These three CHVs were then replaced by community selection. Therefore at the end of the study there were 43 community-selected and 39 self-selected CHVs. The self-selected CHVs tended to be a slightly more educated group compared to the community-selected group with fewer having attended only primary school. Both groups had similar previous experience as CHVs. All other demographic data were comparable between community-selected and self-selected groups of CHVs. CHVs that self-selected fell into two groups: confident experienced CHVs/VHTs and those with little experience who were interested in this area.

**Table 3:** Information about the CHVs in the study

CHV Data	Control *n* = 39		Intervention *n* = 43	
	No.	%	No.	%
Mean age (years)	39		41	
Males	17	42 %	22	51 %
Females	17	58 %	21	49 %
Born in the area	32	84 %	35	81 %
Selection				
Community selected	20	51 %	24	44 %
Self-selected	19	49 %	19	56 %
Educational level community selected CHVs				
Primary	4	20 %	9	38 %
Secondary	14	70 %	12	50 %
Higher	2	10 %	3	13 %
Educational level Self Selected CHVs				
Primary	1	5 %	5	26 %
Secondary	17	90 %	11	58 %
Higher	1	5 %	3	16 %
No. years as a CHV				
Community selected	5.5		7.3	
Self selected	7.2		2.1	
Religion				
Muslim	16	42 %	13	30 %
Christian	22	58 %	30	70 %
Marital status				
Married	33	87 %	39	91 %
Un-marrried	4	10 %	4	9 %
Divorced or widowed	1	3 %	0	0 %
Children				
Number	6.8		5.9	
No children	2	5 %	2	12 %
Work				
Housework	12	32 %	15	35 %
Farmer	27	71 %	27	68 %
Shopkeeper	6	16 %	1	2 %
Leader	13	26 %	13	30 %
Teacher	1	3 %	7	16 %
Other	0	3 %	3	7 %

**Table 4:** Household survey outcomes

	Baseline		Post		% change^a^
	No.	%	No.	%	
Outcome 1: Woman knows a CHV					
Control	57	52 %	75	74 %	22 %
Intervention	61	58 %	92	92 %	34 %
Difference between control and intervention 18 % (*p* = 0.08)					
Outcome 2: Woman knows the name of a CHV					
Control	50	45 %	68	67 %	22 %^
Intervention	56	53 %	82	82 %	29 %
Difference between control and intervention 15 % (*p* = 0.11)					
Outcome 3: Woman has been visited by a CHV					
Control	46	42 %	64	63 %	21 %
Intervention	40	40 %	81	81 %	41 %
Difference between control and intervention 18 % (*p* = 0.11)					
Outcome 4: Children who had diarrhea in the past two weeks and were					
given ORS	*n* = 22		*n* = 33		
Control	16	73 %	29	89 %	16 %
Intervention	*n* = 22		*n* = 34		
	15	68 %	31	90 %	22 %
Difference between control and intervention 1 % (*p* = 0.96)					
Outcome 5: Woman has a functioning tippy taps					
Control	7	6 %	33	35 %	24 %
Intervention	5	5 %	47	47 %	42 %
Difference between control and intervention 12 % (*p* < 0.002)					

^a^Change from baseline

**Table 5:** CHVs utilizing health apparatus at home

Villages	Tippy taps				Dish racks			
	Pre		Post		Pre		Post	
	No.	%	No.	%	No.	%	No.	%
Control *N* = 38	5	13 %	33	86.8 %	20	52.6 %	37	97.3 %
Intervention *N* = 43	6	14 %	36	83.7 %	22	51.1 %	40	93 %

### Process measures

Overall there was a trend for the CHVs who received supportive supervision to make more home visits particularly to pregnant women and newborn babies and to be better known in the community. While the difference was not significant, possibly due to sample size, 81 % of women had been visited during the past year by a CHV in the intervention villages compared to 63 % in the control villages (*p* = 0.11) and 87 % of respondents in the intervention village and 67 % in the control villages knew a CHV by name (*p* = 0.11).

Early in the study it became evident that many community members believed that insecticide treated nets for malaria prevention, family planning and immunization were intended to cause infertility or ‘kill their children’. Additionally a number of cultural beliefs and scientific misunderstandings were identified related to pregnancy, childbirth and family planning. Therefore dealing with issues of developing trust in the community and developing the confidence of the CHVs to make home visits to address concerns was critical to success, even for experienced CHVs. This resulted in the educational component related to home visits to pregnant women and newborn babies being delayed and coming toward the last 4 months of the research. As such, it was difficult to detect significant differences however there was a positive trend in the intervention villages of increased visits to both of these groups.

Prior to the study commencing 2 % of pregnant women were visited once by a CHV in the control villages while 33 % of pregnant women were visited an average of 2.3 times by a CHV during their pregnancy. In the intervention villages 9 % of pregnant women were visited once during the year prior to the study while 46 % of women were visited an average of 3.5 times during the study. Similarly 28 % of newborns in the control villages were visited once in the year prior to the study while 44 % of newborns were visited an average of 1.8 times in the year of the study. This was compared to 9 % of newborns in the intervention group who received one visit prior to the study and 67 % of newborns receiving an average of two visits in the year of the study (*p* = 0.74). These visits were likely made in the last 3 to 4 months of the study after the CHVs learnt about making home visits to pregnant women and newborns.

The CHWs and CHVs reported that having both men and women as educators was important for engaging men in reproductive health issues. The men were able to encourage husbands and partners to go with their wives to antenatal visits and for testing for sexually transmitted infections, including HIV, to ensure that as part of the birth planning process funds were available for clinic visits and institution based births as well as for emergencies.

### Outcome measures

There was an improvement from baseline in all outcome indicators, except for the number of dish racks, in both intervention and control villages. At baseline there were 54 % of the homes with dish-racks and by the end of the study the 66 % and 46 % of homes in the control and intervention villages respectively had dish-racks. Training emphasis was placed on tippy taps rather than dish racks, because at baseline only 5 % of all homes visited had a functional tippy taps and having access to a source for hand washing was considered important especially for newborn care. After 1 year significantly more functioning tippy taps (*p* < 0.002) were present in the intervention villages (47 %) than in control villages (35 %). Use of ORS during diarrhea was already very high before the intervention in villages both intervention and control (see Table 3) and so, not surprisingly, there was no difference (*p* = 0.96) in use of ORS at follow-up.

The CHVs themselves implemented a number of the interventions in their own homes and were examples in the community. 52.6 % (*n* = 20) and 51.1 % (*n* = 22) of CHVs in the control and intervention villages respectively had a dish-rack at baseline while 97.3 % (*n* = 37) and 93 % (*n* = 40) respectively had a dish-rack at the end of 1 year. Similarly, 13 % (*n* = 5) of the control CHVs and 14 % (*n* = 6) of the intervention village CHVs had a tippy tap at the beginning of the study and 86.8 % (*n* = 33) and 83.7 % (*n* = 36) of the control and intervention CHVs respectively had tippy taps in their own homes by the end of the study. This was far higher than the control and intervention households that had tippy taps at the end of the year and showed CHV potential to act as positive examples.

## Discussion

The search for effective supervisory models for CHVs working to educate families about reproductive health is ongoing. Supervision needs to be practical, supportive and cost effective, and questions remain on who should undertake this role, particularly with the upsurge of full-time CHWs being integrated into the health system. Having a clear understanding of the roles of both the CHVs and CHWs – which will differ from program to program – is important for those supervising and those being supervised.

While this study was a proof of concept, and too small to expect to show significant differences between the groups, it contributes, in part, to this body of knowledge. The role of the CHV was to make home visits to pregnant women and newborn babies and to educate families on family planning and on ways to improve sanitation and hygiene.

The intervention made a statistically significant difference to use of tippy taps and all other differences, while not statistically significant, favoured the intervention villages.

The predominant outcome of the study was related to improved community trust, participation and cooperation, which resulted in a statistically significant increase in the number of tippy taps that were built in a neighborhood and a safe environment to discuss what were, at times, difficult reproductive health issues. While not significantly compared to the control groups, there was an overall increased numbers of visits to neighbors and high level coverage was made possible by having pairs of CHVs, one male and one female, working together with approximately 50 homes. Pairing of men and women was found to be an effective strategy for engaging men in reproductive health processes and allowing them to discuss their fears and concerns with other men. A realistic vision of the time a volunteer could give (5 to 10 h per week) was set and all the work and training were within walking distance of the CHVs homes in an attempt to reduce costs. This departs from the current Ugandan Ministry of Health strategy, focusing on two part-time CHVs per village who are given centralized training. Balancing ratios of households to be visited with realistic time and workloads for CHVs, who are self supporting, needs to be taken into consideration if they are reach the entire village particularly on foot.

On comparison with the usual practice of CHV training and supervision in Uganda, the control group received high quality monthly training in their own villages rather than intermittent training and centralized quarterly meetings if supervised. This might explain why the indicators improved from baseline in both the intervention and control groups. The supportive supervision/accompaniment intervention might have resulted in bigger follow-up differences from the control group if they had received the usual training available to the CHVs in Uganda.

Having four full-time CHWs in the study proved to be worthwhile. With regular training and reflection spaces, two CHWs were able to provide training and supportive supervision to approximately 40 CHVs working with a population of 5000 inhabitants of four villages. They were also able to form relationships in the community, with leaders, NGOs and clinics. That the CHWs did not all have a strong health background did not seem to detract from increased CHV activity. In reality, having non-professional CHWs might have been important in creating an atmosphere of supportive supervision and accompaniment as no one felt superior, and all were learning together.

The willingness of the CHVs to implement what they learnt in their own homes was also important. There are some areas of overlap in the model proposed in this study to the earlier-mentioned approach taken with model families and CHVs who are trained and supervised by Health Extension Workers (HEWs) in Ethiopia [3, 7–9]. While detailed information is not available across the program, mention is made of two HEWs working with 50 or 60 model families. Through this approach a ripple effect can be achieved as more and more community members are engaged in health improvement strategies to improve maternal and child health outcomes [3, 7–9].

Development of a model that combines full-time CHWs who then work with a number of CHVs in a village would seem to enable good coverage by the volunteers and the development of capabilities that would enable them, in the longer term, to be increasingly more effective in well-circumscribed areas of reproductive health education and action. Although it may be logistically easier to have supervisors that are already employed as part of the clinical workforce, it is likely that full-time CHWs that understand the role of the CHV and the reality of the community through an ongoing relationship will more likely be effective. In the current study only one of the four CHWs resided in Budondo. This limited what they were able to do in the community. A working model of CHWs who offer clinical and preventative services such as basic antenatal care, integrated Community Care Management (iCCM), family planning and immunization in the villages, as well as train and accompany the CHVs could not be fully utilized and remains an area for further research.

Elements of effective supervision in the study described in this paper included: consistency (monthly), a non-threatening approach and, relevance to the training the CHVs had just received. The CHWs and the CHVs learnt together and reflected on their learning. Additionally, the CHVs were able to implement what they had learnt in their own homes, hence acting as role models. Collaboration with the local leaders was also strong, with a number of the CHVs being community leaders themselves.

Policy makers and planners interested in having CHVs extend the work of their full-time CHWs would need to ensure that a fundamental role of CHWs was to train and supportively supervise the CHVs. Additionally, CHVs may need T-shirts or name badges, educational flip-charts and bags – for example – without which community trust and acceptance may be difficult to build [1, 2, 6, 34]. The ratio of CHWs to CHVs will also need to be established and may depend on issues such as population density, rain fall, roads and general terrain where the CHVs are working. Some examples in the literature of programs that have been exploring creative and cost effective supervisory models include the HEW training and supervision model in Ethiopia [3, 7–9], the peer support model in Rwanda [35], the accompaniment model of Partners in Health [31] that builds capacity of CHW supervisors from strong experienced CHWs and the Healthy Child Uganda [10, 32] model that has initial close supervision which reduces as capacity is built in the community. Having a dedicated cadre of individuals for community-based clinical, preventative and transformative action capable of maximizing their reach through community engagement seems important if the benefits inherent in this approach for reproductive health outcomes are to be realized.

## Strengths and limitations of the study

A cluster design was chosen because the intervention was structured around communities rather than individuals. We chose the village as the cluster unit of randomisation for the following reasons: it is a standard geopolitical unit and village leaders were key points of liaison. Discussions with local people suggested that randomisation at village level with a one village buffer would avoid contamination.

A major limitation is the small number of randomised villages and the small number of survey participants, resulting in low statistical power of the study. It is possible that more differences at follow-up in process and outcome measures between intervention and control villages would have been statistically significant if the study had involved a larger number of villages. A larger study could also have assessed the impact of the CHV intervention on maternal and newborn outcomes.

Another limitation is that the intervention was only for 1 year. The time taken for initial training and accompaniment centered on gaining community trust, community mapping, action plans and hygiene was both a strength and a weakness. Education related to making two visits to a pregnant woman and three visits to a newborn baby in the first week took place 6 to 8 months into the intervention and would therefore have limited the number of mothers and newborns visited. Nonetheless the results are a promising proof of concept of the impact that training and accompaniment can have on CHV activity in a community. Additional follow-up would be required to understand the true attrition rates after the intensity of the initial 1-year training and accompaniment intervention reduced. This was not within the scope of the current study.

## Conclusion

Decisions about accessing reproductive health are made within the context of a family and may be determined by cultural norms and scientific misconceptions. Having a team of 2 CHWs to 40 CHVs enables close to community access to information, conversation and services. While CHWs have been found to improve maternal and newborn health outcomes, finding a sustainable model to offer quality supportive supervision has remained elusive. This study suggests that regular training of CHVs at village level by full-time CHWs plus supportive monthly accompaniment improves reproductive health related activity by the CHV and the community in villages in rural Uganda. In this setting, supportive supervision was undertaken by individuals who were familiar with the role of the CHV and able to learn with them as they overcame challenges that helped to build trust in the community. Having realistic expectations of the time that a CHV can give and distances they can cover are also important. CHVs acted as role models in the community by applying what they learnt. Combining full-time CHWs and volunteer CHVs, both male and female, both of whom receive quality training and supervision, appears to be a promising strategy that addresses community member concerns, increases coverage and engagement of community members in reproductive health activities.
